# Resveratrol as a multitarget modulator in diabetic retinopathy: a systematic review of in vitro and in vivo studies

**DOI:** 10.1186/s12886-026-04623-0

**Published:** 2026-01-29

**Authors:** Ohisa Harley, Yufilia Suci Amelia, Elsa Gustianty, Nanny N. M. Soetedjo, Arief S. Kartasasmita

**Affiliations:** 1 Doctoral Program in Medical Sciences, Faculty of Medicine, Padjadjaran University, Ir. Soekarno Street Km 21, Bandung, West Java Indonesia; 2 Netra Eye Clinic Centre, Sumatera Street No. 46-68, Bandung, West Java Indonesia; 3 Unpad Hospital, Cirebon – Bandung Raya Street, Jatinangor, West Java Indonesia; 4 Departement of Ophthalmology, Faculty of Medicine, Padjadjaran University, Ir. Soekarno Street Km 21, Bandung, West Java Indonesia; 5 Cicendo National Eye Hospital Center, Bandung, West Java Indonesia; 6 Departement of Endocrinology and Internal Medicine, Faculty of Medicine, Padjadjaran University, Bandung, West Java Indonesia

**Keywords:** Resveratrol, Diabetic retinopathy, Oxidative stress, Microglia, Inflammation

## Abstract

**Background:**

Resveratrol (RSV) has been extensively investigated for its antioxidant and anti-inflammatory properties across various disease settings; however, its upstream mechanisms in diabetic retinopathy (DR) remain insufficiently characterized. Given that early DR involves oxidative stress, mitochondrial disruption, and microglia-associated inflammatory amplification, this systematic review aims to synthesize preclinical evidence on how RSV influences these early molecular and cellular events in diabetic retinal models.

**Methods:**

A systematic search of PubMed, EBSCO, and ProQuest identified in vitro and in vivo studies examining RSV in diabetes-induced retinal disease. Eligible studies evaluated mechanistic outcomes related to oxidative stress, inflammation, mitochondrial function, or neurovascular integrity. Risk of bias was assessed using SYRCLE’s tool for in vivo studies and QUIN-based criteria for in vitro studies.

**Results:**

Twenty studies met inclusion criteria. RSV consistently activated SIRT1 and improved mitophagy and mitochondrial dynamics, leading to reduced ROS-mediated inflammatory activation. RSV also modulated apoptotic pathways by suppressing caspase activity and enhancing SIRT1/PGC-1α–associated survival signalling. Antioxidant defences were strengthened through Nrf2/HO-1 activation, increasing endogenous antioxidant capacity and lowering oxidative injury markers. The most prominent and consistent finding was RSV’s strong anti-inflammatory effect, characterized by reduced TNF-α, IL-6, and IL-1β, inhibition of NF-κB and HMGB1 signaling, and attenuation of microglia-driven inflammatory amplification, supporting a shift toward an anti-inflammatory signaling profile. In vivo studies generally used daily doses ≥ 10 mg/kg, with longer durations producing more consistent neuroinflammatory improvement.

**Conclusion:**

RSV exerts broad protective actions in DR by modulating mitochondrial function, inflammation, oxidative stress, and angiogenic signaling. With its upstream, multitarget profile, RSV represents a promising adjunctive or early-stage therapeutic candidate. Clinical studies are needed to establish optimal dosing, delivery methods, and translational efficacy in human DR.

**Clinical trial number:**

Not applicable.

**Supplementary Information:**

The online version contains supplementary material available at 10.1186/s12886-026-04623-0.

## Introduction

Diabetic retinopathy (DR) is among the most common microvascular complications of diabetes mellitus and remains a leading cause of preventable blindness in working-age adults [1, 2]. Over one-third of individuals with diabetes are expected to develop some degree of retinopathy, with the global burden increasing alongside diabetes prevalence [1, 3]. Current treatments, such as anti-VEGF therapy and laser photocoagulation [4], are effective primarily in later stages of disease and do not address the early neuroinflammatory and metabolic disturbances that initiate retinal damage. This has led to growing interest in upstream-acting agents capable of targeting early pathogenic events.

Emerging evidence shows that DR is not solely a vascular condition but also involves early oxidative stress, microglial activation, mitochondrial disruption, and neuroglial dysfunction [5–7]. These insights underscore the need for therapeutic approaches that act before irreversible neurovascular injury occurs.

Among naturally derived compounds, resveratrol (RSV) has attracted increasing attention in DR research due to its pleiotropic biological activities [8, 9]. As trans-resveratrol represents its biologically active isomer, RSV exerts effects beyond conventional antioxidant action. Unlike many dietary polyphenols that primarily act as free radical scavengers, RSV modulates upstream regulatory pathways via SIRT1-linked deacetylation and HMGB1-related inflammatory signaling, positioning it as a multitarget regulator rather than a single-pathway antioxidant [10, 11]. 

Although RSV has been extensively investigated in metabolic, vascular, and inflammatory disorders [8, 12–14], existing reviews in DR predominantly emphasize its downstream antioxidant or anti-angiogenic effects. In contrast, its upstream roles in early DR—particularly in neuroinflammatory regulation, microglial activation and polarization, mitochondrial quality control, and epigenetic-associated signaling—remain underexplored. Therefore, this systematic review provides current in vitro and in vivo evidence to clarify the role of RSV in modulating several targets —microglia-driven inflammation, mitochondrial stress responses, and early neurovascular alterations— during the initial stages of DR [8, 9, 12]. 

## Methods

### Protocol and registration

This systematic review was carried out according to the Preferred Reporting Items for Systematic Review and Meta-analysis (PRISMA) Guidelines and registered in PROSPERO (CRD420251145127).

### Eligibility criteria and outcomes of interest

Research studies could be considered for inclusion if they met the following criteria.

1. Population: Diabetic induction (in vitro and in vivo).
2. Intervention/Exposure: Diabetic animal with RSV.
3. Control: Control, diabetic animal without RSV.
4. Outcome: Mechanism of RSV in retina.
5. Designs: experimental and animal study.

For exclusion criteria were as follows: (1) RSV derivates and (2) combination RSV with other drugs.

### Search strategy

We used medical subject headings (MeSH terms and free-text keywords associated with diabetic retinopathy and systemic blood markers to locate relevant research. Multiple databases, such as PubMed, EBSCO, and ProQuest, were searched. The search strategy included the following terms: ((“resveratrol“[MeSH Terms]) OR (“Resveratrol“[Text Word])) AND ((“diabetic retinopathy“[MeSH Terms]) OR (“diabetic retinopathy“[Text Word])). We also manually reviewed the reference lists of the included research and relevant reviews, and searched Google Scholar to find any potentially relevant articles. (see Supplementary File 1). We limited our search to articles published in English and with full text.

### Data selection, collection, and extraction

We organized the identified studies using Mendeley reference manager. First, duplicates were removed, followed by screening titles and abstracts to determine eligibility. Two authors (OH and YSA) independently conducted this screening. Any disagreements during selection or quality assessment were discussed with other authors (EG, NS, ASK). Relevant data were extracted, including author, year, study design, intervention or treatment, resveratrol dose, and findings, which were summarized in a table.

### Risk of bias assessment

We conducted SYRCLE’s risk of bias assessment for in vivo studies and QUIN risk of bias tools assessment for in vitro studies.

## Results

### Characteristics of the included study

**Fig. 1:**
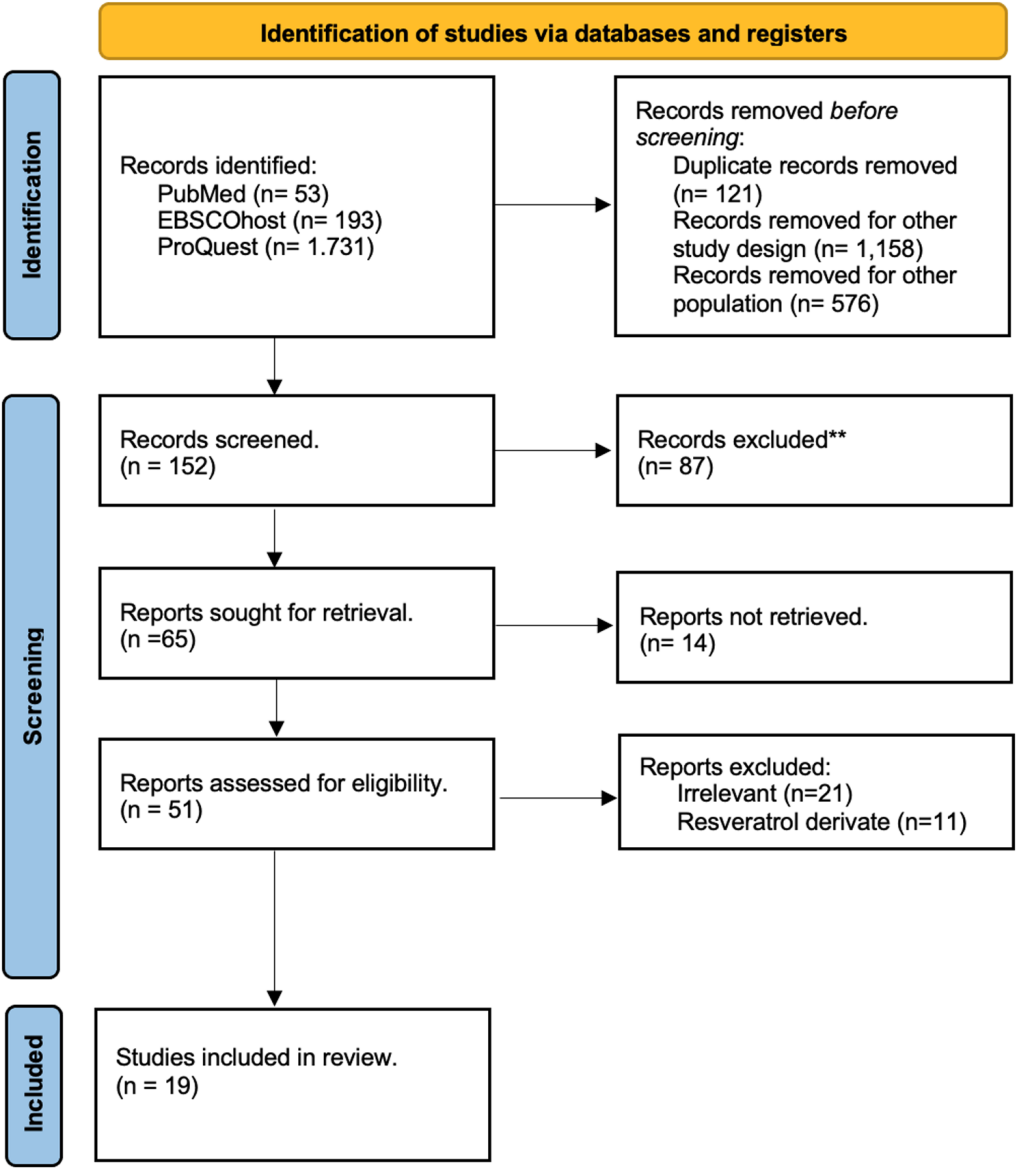
PRISMA flow diagram

A total of 1,977 studies were identified through database searches and manual exploration (Fig. 1). After removal, 51 studies underwent initial screening based on their titles and abstracts. Out of these, 51 studies were further assessed to determine their eligibility criteria. A total of 20 studies were included in this review, comprising 15 in vitro and 13 in vivo experimental models (Tables 1 and 2).

**Table 1:** In vitro studies of resveratrol in retina

Author, Year	Origin	Cells	Insult	RSV Concentration	Laboratory Technique	Findings and Mechanism of RSV	Function
Alka K, 2023 [6]	N/A	HRECs	High glucose (20 mM D-glucose)	25 µM	CCK-8 assay, qRT-PCR, immunostaining, mitophagy dye flow cytometry, immunofluorescence, Western blot	- Decreased Mfn2 acetylation, increased GTPase activity, reduced mitochondrial fragmentation, and enhanced mitophagy flux via SIRT1 activation - Restored mitochondrial fusion and removal of damaged mitochondria.	- Restore mitochondrial function
Chang, 2017 [28]	Human cell line	ARPE-19	Hypoxic (CoCl2 100 to 1000 µM)	20 µM	Immunofluorescent, Immunoprecipitation, Western blot, gelatin zymography, MTT assay, RT-qPCR, and ELISA	- Reduced inflammation through increased SIRT1 leading to decreased HMGB1 acetylation and suppression of TLR4/RAGE signaling (inflammation receptors). - Anti-Angiogenic and anti-fibrogenic factors via inhibition of the Akt/p38/NF-κB pathway. - Maintained the retinal function in proliferation and migration cells	- Anti-inflammation - Anti-angiogenesis
Chen Y, 2019 [16]	Primary cell	RREC	High glucose (30 mM D-glucose)	10–500 µM	CCK-8 assay, qRT-PCR, western blot, ELISA Kit for inflammatory retina	- Increased cell viability and reduced apoptosis by suppressing cleaved caspase-3 expression. - Enhanced PON1 activity resulted in decreased levels of IL-1β, IL-6, TNF-α, VEGF, IFN-γ, and MCP-1.	- Reduced apoptosis - Anti-inflammation
Giordo, 2021 [24]	Primary cell	HERCs	High glucose (30 mM D-glucose)	1 µM	qRT-PCR, ELISA, ROS assay, cell apoptosis assay, MTT assay	- Reduced ROS levels and prevented EndMT via the PKC/NOX2 pathway - Restored endothelial marker expression (CD31, CDH5, vWF) and reduced mesenchymal markers (α-SMA, vimentin, collagen I) - Inhibited apoptosis	- Anti-oxidant - Reduced apoptosis
Lee, 2022 [25]	Human adult RPE	ARPE-19	High glucose (30 mM D-glucose)		SA-β-gal staining. western blot, confocal microscopy, ROS assay, RT-qPCR	Protected retinal cells from high glucose–induced damage via the SIRT1 pathway, with decreased p53/p21 expression and reduced cellular senescence and oxidative stress	- Anti-oxidant
Li, 2017 [17]	Primary bovine cell culture	BRECs	High glucose (30 mM D-glucose)	1, 5, 10, 20 µM	Cell viability assay, peroxide-sensitive fluorescent probe, flow cytometry, RT-PCR, transfection, western blot	Inhibited apoptosis and ROS production via activation of the AMPK/SIRT1/PGC-1α pathway	- Reduced apoptosis - Anti-oxidant
Liu S, 2016 [29]	Peripheral blood from PDR and idiopathic macular ERM patients	PBMCs	N/A	10 µM	ELISA, RT-qPCR, dan western blot	Activated SIRT1 and reduced IL-17 expression in PDR.	- Anti-inflammation
Liu, 2020 [32]	Primary cell obtained from Cell Systems Corporation	HRCECs	High glucose (25 mM D-glucose)	10 µM	SA β-gal, qRT-PCT, western blot, immunofluoresence	- Prevented mitochondrial dysfunction by suppressing miR-1, miR-19b, and miR-320a, leading to increased SIRT3, SIRT4, and SIRT5 expression - Inhibited endothelial dysfunction and EndMT by preserving CD31 and VE-cadherin expression and reducing SM22 and vimentin levels - Reduced cellular stress, as indicated by decreased SA-β-gal staining - Downregulated VEGF expression, thereby attenuating retinal damage	- Restored mitochondria function - Anti-oxidant - Anti-angiogenesis
Liu, 2021 [18]	Human RPE cells from from Zhongqiaoxinzhou Biotech	ARPE-19 cells	High glucose (30 mM D-glucose)	30 µM	MTT, ROS and apoptosis assay, VEGF and anti-oxidant assay, RT-PCR, transfection, western blot	Reduced oxidative stress and inflammatory cytokine expression (IL-6, TNF-α, and NF-κB) via the AMPK/SIRT1 pathway Reduced apoptosis via p53 suppression	- Anti-inflammation - Anti-oxidant - Reduced apoptosis
Peng Y, 2025 [23]	Primary cell	HRCECs	High glucose (30 mM D-glucose)	5, 10 and 20 mM	Flow cytometry, TUNNEL, tube formation assay, ROS assay, ELISA, qRT-PCR, western blot	- Reduced inflammation and oxidative stress, characterized by decreased IL-1β levels (significant at 20 mM) - Activated the SIRT1/HMGB1 pathway, leading to suppression of inflammation (IL-1β, IL-6, TNF-α), oxidative stress, and ferroptosis - Exerted anti-angiogenic effects through reduced VEGF expression	- Anti-inflammation - Anti-oxidant - Anti-angiogenesis
Wang, 2020	Mouse	BV-2 microglial cells	1 µg/mL LPS	10 µM	cytotoxicity assay, Nitrite assay (Gries Assay), qRT-PCR, ELISA, Western Blot	- Inhibited nitric oxide (NO) production in LPS-stimulated cells - Reduced pro-inflammatory cytokine expression - Inhibited NF-κB and MAPK activation - Promoted microglial polarization from the M1 to the M2 phenotype	- Anti-inflammation - Microglia polarization
Wang Y, 2025 [15]	Sprague-Dawley rats at postnatal (PN) days 5 to 7	Primary culture of retinal Müller cells	High glucose (25 mM D-glucose)	20 mM	Viability cell, Gen and protein expression, MDA, GSH, Fe²⁺ assay	- Exerted antioxidant effects by decreasing MDA levels and increasing GSH content - Regulated Nrf2 and related signaling pathways to inhibit ferroptosis	- Anti-oxidant
Wan Z, 2025 [22]	Sprague-Dawley rats at postnatal days 5 to 7	Retina Muller Cells	High glucose (25 mM D-glucose)	20 mM	transmission electron microscopy, qRT-PCR, western blot, TUNEL analysis, immunocytochemistry	- Increased viability of high glucose–cultured retinal Müller cells (RMCs) - Modulated dysregulated miR-29b/SP1–mediated apoptotic signaling - Reversed autophagy inhibition	- Reduced apoptosis
Yuan, 2024 [19]	C57BL/6 mouse	primary retinal ganglion cells (RGCs)	High glucose (25 mM D-glucose)	10 nM	qRT-PCR, oxidative assay (SOD and MDA)	- Upregulated the Nrf2/HO-1 signaling pathway and downregulated caspase-3–mediated apoptosis - Reduced oxidative stress, as indicated by decreased MDA levels and increased SOD activity	- Reduced apoptosis - Anti-oxidant
Zeng K, 2017 [21]	Sprague-Dawley rats at postnatal days 5 to 7	Retina Müller Cell	High glucose (25 mM D-glucose)	10, 20, 30 mM	Annexin V/PI staining, flow cytometry, qRT-PCR, western blot, caspase-3 assay	- Exerted anti-apoptotic effects by suppressing SP1, caspase-3, and Bax expression, while upregulating Bcl-2 and the miR-29b pathway	- Reduced apoptosis
Zeng K, 2016 [20]	Sprague-Dawley rats at postnatal days 5 to 7	Retina Müller Cell	High glucose (25 mM D-glucose)	10, 20, 30 mM	Glutamate uptake assay, Immunocytochemistry, qRT-PCR, western blot	Enhanced glutamate uptake by increasing glutamine synthetase (GS) activity and GLAST expression in Müller cells	- Reduced the Glutamate

EndMT: Endothelial-to-Mesenchymal Transition; ERM: Epiretinal Membrane; PBMCs: peripheral blood mononuclear cells; PDR: Proliferative Diabetic Retinopathy; BREC: Bovine retinal capillary endothelial cells; GS: Glutamine synthase; GLAST: glutamate transporters; HO-1: heme oxygenase-1; GSH: Glutathione; MDA: Malonic Dialdehyde; HRCECs: Human retinal Capillary Endothelial Cells; LPS: lipopolysaccharide; MDA: malon dialdehyde; Nrf2: nuclear factor erythroid 2-related factor 2; RREC: Rat retinal endothelial cell; SA β-gal: Senescence-associated staining β -Galactosidase; SOD: superoxide dismutase

**Table 2:** In vivo studies of resveratrol in diabetic rats

Author, Year	Animals	Tissues	Induction	RSV Dose	Laboratory Technique	Mechanism	Function
Alka, 2023 [6]	C57BL/6J mice	Retina	Injection of STZ 55 mg/kg; for 4 days; intravenous	N/A	GTPase activity assay, Western blot, ELISA, flow cytometry, immunofluorescence	- Reduced Mfn2 acetylation, restored GTPase activity, and increased autophagosome formation and mitophagy via SIRT1 overexpression, thereby preventing mitochondrial damage	- Restored mitochondrial function
Al-Hussaini, 2021 [27]	Dark Agouti rats	RPE	Injection of STZ 50 mg/kg; single dose; intraperitoneal	5 mg/kg/day per oral for 30 days	RT-PCR, western blot, oxidative assay	- Reduced oxidative stress and apoptosis - Normalized apoptosis-related protein expression (caspase-3 and caspase-9) - Modulated the MAPK/ERK/JNK signaling pathway	- Antioxidant - Reduced apoptosis
Chen Y, 2019 [16]	Sprague-Dawley rats	Retina	Injection of STZ 60 mg/kg; single dose; intravenous	0.1 µg/mL or 1 µg/mL, single dose, intravitreal injection 5, 10 atau 50 µg/kg/days for 12 weeks via tail vein injection	Evans blue assay, blood sampling analysis, western blot, AGE ELISA kit, TUNEL staining	- Maintained retinal permeability - Increased PON1 activity and reduced inflammatory mediators (IL-1β, IL-6, TNF-α, VEGF, IFN-γ, and MCP-1) - Reduced apoptosis - Decreased oxidized LDL (ox-LDL), thereby attenuating oxidative stress	- Anti-inflammation - Reduce apoptosis - Antioxidant
Fathalipour, 2019 [26]	Spargue-Dawley rats	Retina	Injection of STZ 60 mg/kg intravitreal (five injections at three days interval)	10 µM intravitreal	Oxidative assay (SOD and IPF2α), western blot, Hematoxylin-eosin staining	- Reduced oxidative stress by increasing SOD activity and AKT signaling and decreasing retinal p-ERK levels - Preserved retinal thickness, ONL, and RGC integrity - Maintained retinal vascular permeability	- Antioxidant - Maintained the retinal structure
Liu, 2020 [32]	C57BL/6J mice	Retina	Injection of STZ 50 mg/kg/day for 5 consecutive days; intraperitoneal	N/A	qRT-PCT	- Significantly decreased SIRT3 and SIRT5 expression, with no significant change in SIRT4	- Anti-oxidant
Mohammad, 2022 [30]	Sprague Dawley rats	Retina	Injection of STZ 55 mg/kg	50 mg/kg; two times per 4 weeks; intraperitoneal	RT-PCR, western blot, FITC-dextran assay	- Increased SIRT1 expression - Reduced HMGB1 and RAGE expression - Maintained blood–retinal barrier (BRB) integrity	- Anti-inflammation - Anti-oxidant
Peng Y, 2025 [23]	c57 mice	Retina	Injection of STZ 50 mg/kg/day for 5 consecutive days; intraperitoneal	20 mg/ kg RES once a day for 4 weeks via oral gavage	Flow cytometry, TUNNEL, tube formation assay, ROS assay, ELISA, histology, qRT-PCR, western blot	- Exerted anti-inflammatory and anti-angiogenic effects via the SIRT1/HMGB1 pathway - Reduced HMGB1 acetylation and decreased VEGF, IL-1β, IL-6, and TNF-α expression - Preserved endothelial function, as indicated by maintained CD31 expression	- Anti-inflammation - Anti-angiogenesis
Wang Y, 2025 [15]	Sprague–Dawley and Nrf2 knockout diabetic mice	Retina	Injection of STZ 35 mg/kg; intraperitoneal	10 mg/kg/d for 12 weeks via oral gavage	ERG, TEM, western blot, qPCR, immunohistochemical, MDA, GSH, Fe²⁺ assay	- Improved retinal function, as demonstrated by electroretinography (ERG) - Improved mitochondrial morphology in retinal INL cells - Enhanced antioxidant capacity by decreasing MDA levels and increasing GSH content - Regulated Nrf2 and related signaling pathways to inhibit ferroptosis - Reduced PTGS2 expression	- Improved retinal function in ERG - Restored mitochondrial function - Anti-oxidant
Wan Z, 2025 [22]	Sprague-Dawley Rats	Retina Muller Cells	Injection of STZ 35 mg/kg; intraperitoneal	10 mg/kg/day via oral gavage	ERG, transmission electron microscopy, qRT-PCR, western blot, immunocytochemistry	- Improved retinal neurofunction and restored autophagic activity in diabetic rats	- Improved autophagy
Yuan, 2024 [19]	C57BL6 mice	Retina	Injection of STZ 50 mg/kg/day for 5 consecutive days; intraperitoneal	10 mg/kg/day via oral gavage	ERG, TUNEL staining, immunofluorescent, qRT-PCR, western blot	- Improved retinal morphology and function, as assessed by ERG, accompanied by reduced retinal ganglion cell (RGC) apoptosis	- Improved retinal function in ERG
Zeng K, 2017 [21]	Sprague-Dawley Rats	Retina	Injection of STZ 60 mg/kg; intraperitoneal	5 and 10 mg/kg/day for 1–7 months via oral gavage	TUNEL assay, qRT-PCT immunofluorescence, western blot	- Suppressed apoptotic activity in retinal INL cells, increased miR-29b expression, and downregulated SP1, with greater effects observed at 10 mg/kg RSV	- Reduced apoptosis
Zeng K, 2016 [20]	Sprague–Dawley rats	Retina	Injection of STZ 60 mg/kg; intraperitoneal	5 and 10 mg/kg/day for 1–7 months via oral gavage	ERG, spectrophotometric assay, qRT-PCR, ELISA, western blot	- Reduced blood glucose levels and prevented retinal dysfunction, as assessed by ERG - Preserved ERG amplitudes, including rod a-wave, cone and rod a- and b-waves, and OP2 oscillatory potentials - Upregulated GLAST and glutamine synthetase (GS) mRNA and protein expression	- Improved retinal function in ERG
Zeng K, 2021 [33]	Sprague–Dawley rats	Retina	Injection of STZ 60 mg/kg; single dose; intraperitoneal	5 and 10 mg/kg/day 1–9 months via oral gavage via	TUNEL, immunofluorescence, RT-PCR, western blot	- Reduced retinal apoptosis by approximately 75% across the ONL, INL, and GCL - Suppressed RAX and phosphorylated PKR (p-PKR) protein expression	- Reduced apoptosis

AGE: advanced glycosylated end product; ERG: Electroretinography; GSH: Glutathione; MDA: Malonic Dialdehyde; RGC: Retinal Ganglion Cells; STZ: streptozocin; TUNEL: TdT-mediated dUTP-biotin nick end labelling; qRT-PCR: quantitative real-time polymerase chain reaction

The in vitro studies predominantly utilized human and animal retinal cell lines, including human retinal capillary endothelial cells (HRCECs), retinal pigment epithelial cells (ARPE-19), rat retinal endothelial cells (RREC), primary Müller glial cells, BV-2 microglial cells, and primary retinal ganglion cells. Diabetic insults were most commonly induced using high-glucose conditions (20–30 mM), though some models employed hypoxic stimuli (CoCl₂), lipopolysaccharide (LPS), or oxidative stress inducers. The concentrations of resveratrol used ranged from 1 to 50 µM, with most studies employing doses ≥ 10 µM.

The in vivo studies primarily involved streptozotocin (STZ)-induced diabetic models in Sprague-Dawley rats, C57BL/6 mice, or Dark Agouti rats. Resveratrol was administered either orally, intraperitoneally, or intravitreally, with daily doses ranging from 5 to 50 mg/kg/day over periods of 2 to 12 weeks. These studies evaluated outcomes related to retinal inflammation, oxidative stress, apoptosis, blood-retinal barrier (BRB) integrity, and neurovascular protection. Most in vivo models applied long-term administration to assess sustained effects on retinal structure and molecular parameters relevant to diabetic retinopathy.

### Risk of bias assessment

**Fig. 2:**
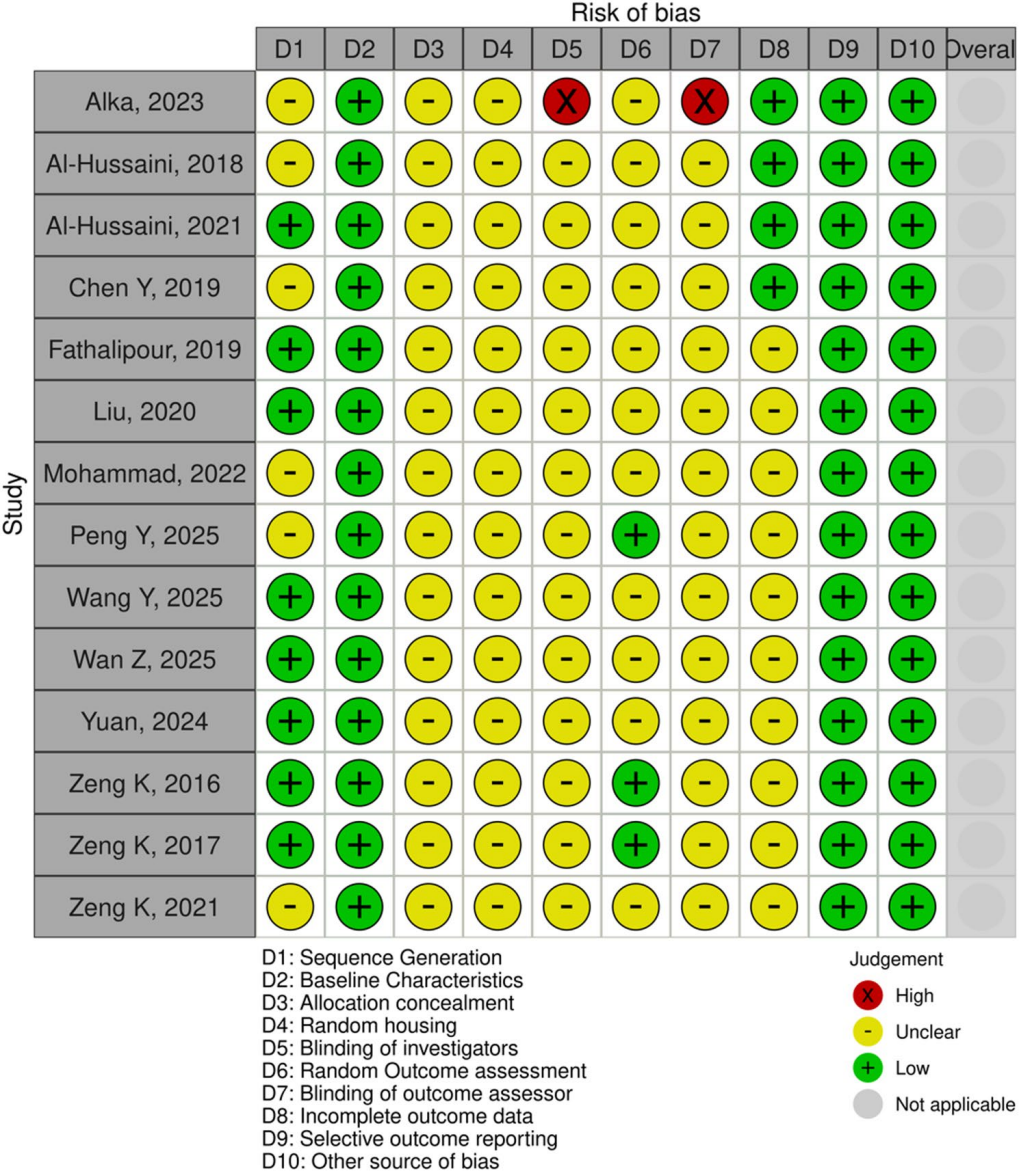
Risk of bias of in vivo studies

**Fig. 3:**
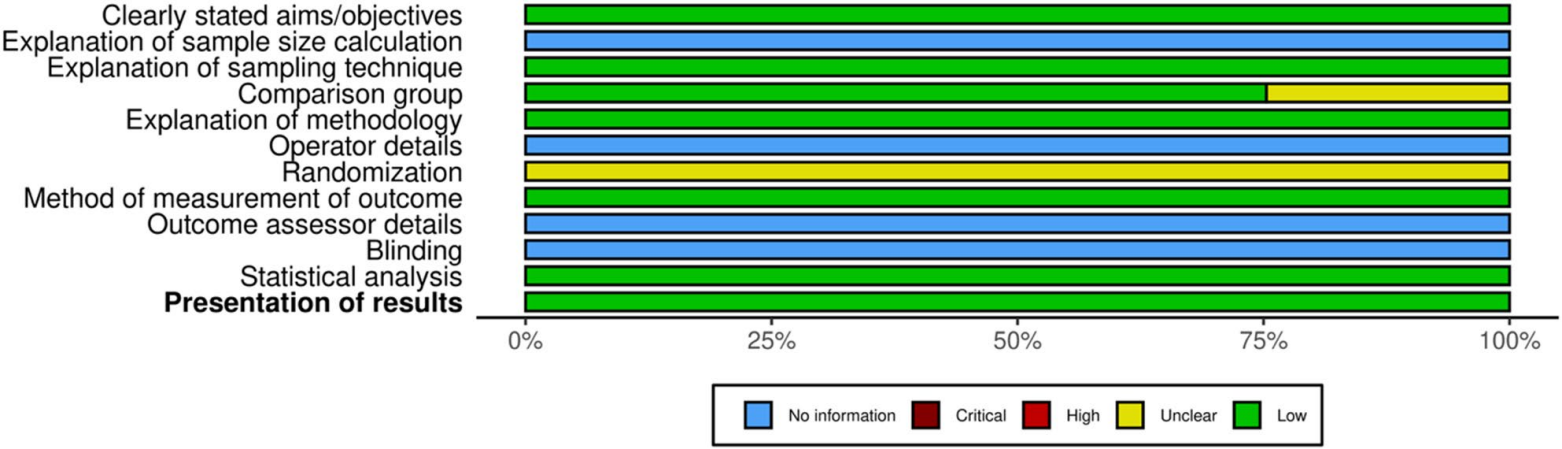
Risk of bias of in vitro studies

The quality assessment of the 19 included studies identifies a moderate risk of bias in in vitro and in vivo studies (Figures 2 and 3). Risk of bias was assessed using SYRCLE’s RoB tool for in vivo studies. As shown in Fig. 2, most studies showed a generally low or unclear risk of bias across the evaluated domains. The majority of studies had a low risk of bias in domains related to outcome data completeness, selective outcome reporting, and other sources of bias. However, several domains, such as random housing, blinding of investigators, and blinding of outcome assessors, were frequently rated as “unclear” due to a lack of reporting.

A complementary assessment using a modified QUIN-based domain summary (Fig. 3) highlighted that only a small proportion of studies justified sample size calculation, sampling technique, and outcome assessor blinding. While most studies clearly stated their aims and described comparison groups and methodology, critical methodological details such as operator identity, randomization procedure, and statistical analysis were often underreported. This indicates a moderate to high concern regarding internal validity and reproducibility in many of the included in vivo studies.

### Resveratrol in diabetic retinopathy

RSV is a naturally occurring polyphenolic compound that has received considerable attention for its antioxidant, anti-inflammatory, and anti-aging properties. Within the context of diabetes-related complications, especially DR, resveratrol has emerged as a multifunctional therapeutic agent at both the cellular and molecular levels. Preclinical studies—both in vitro and in vivo—have consistently demonstrated that RSV exerts multifaceted therapeutic effects in DR. These effects are mediated through key cellular and molecular mechanisms, including the modulation of oxidative stress, mitochondrial dysfunction, inflammation, endothelial barrier breakdown, and pathological angiogenesis. RSV’s biological activity is closely tied to the regulation of several critical signaling pathways, particularly SIRT1, AMPK, Nrf2, and NF-κB, which together form a comprehensive protective network against DR pathogenesis.

### Key molecular pathways modulated by resveratrol

A critical synthesis point here is that RSV does not act through a single linear pathway but instead orchestrates a multilayered response that touches upon inflammation, oxidative stress, mitochondrial metabolism, and gene regulation.

1. *Mitochondrial Protection and Mitophagy Activation: *Mitochondrial dysfunction is a major contributor to DR progression. In vitro study showed that RSV restored mitochondrial integrity by reducing Mfn2 acetylation and increasing GTPase activity in high-glucose-treated HRECs [6]. In vivo, RSV improved mitophagy and mitochondrial morphology in diabetic mice, preserving retinal energy homeostasis via SIRT1 and Nrf2 signaling [15].
2. *Regulation of Apoptosis: Seven studies reported that RSV* suppresses hyperglycemia-induced apoptosis through the AMPK/SIRT1/PGC-1α axis. In vitro studies observed reduced caspase-3 and p53 expression and increased Bcl-2 in RSV-treated retinal cells [16–22]. They also demonstrated that miR-29b-mediated suppression of SP1, reducing apoptosis in Müller cells. This also confirmed in vivo studies that long-term RSV preserved retinal ganglion cell survival and retinal architecture and normalizes apoptosis protein via the MAPK/JNK Pathway [19].
3. *Antioxidant Defense and Ferroptosis Suppression*: Nine studies demonstrated that RSV activates the Nrf2/HO-1 and PKC/NOX2 pathway, increasing glutathione and reducing lipid peroxidation. in vitro studies reported reduced ROS and MDA levels in high-glucose conditions [15, 18, 19, 23, 24]. It also activates the SIRT1 pathway to reduce oxidative damage [17, 25]. Supported by in vivo studies, RSV reduces oxidative stress via MAPK/JNK pathway and demonstrates increased SOD activity and preserved retinal thickness following intravitreal RSV [26, 27].
4. *Anti-Inflammatory Effects: *Six studies demonstrated that RSV reduces inflammation by inhibiting NF-κB and HMGB1 signaling through increasing SIRT1 and PON1 pathways. In vitro studies found that RSV decreased pro-inflammatory cytokines [18, 23, 28, 29]. This was confirmed in in vivo studies, which showed a reduction in IL-6, TNF-, IL-1β, NF-κB, NO, and MCP-1, and preserved BRB integrity in diabetic rats [16, 23, 30].
5. *Microglia polarization*: A study shows RSV supports the polarization of microglia from M1 proinflammatory phenotypes to M2 anti-inflammatory phenotypes, which reduces inflammation by increasing anti-inflammatory responses effects [31]. Despite the function, RSV is also found to be anti-angiogenic and preserves BRB function. Preservation of Blood-Retinal Barrier (BRB): RSV inhibits EndMT and preserves endothelial integrity through suppression of PKC/NOX2 signaling. In vitro studies showed restored expression of CD31 and VE-cadherin [24, 32]. In vivo studies also demonstrated improved BRB function, retinal layer stability, and preserved endothelial function [16, 23].
6. *Anti-Angiogenic Action: *RSV inhibits angiogenesis by reducing VEGF, bFGF, and TGF-β2 through SIRT1/HMGB1 and NF-κB pathways. Studies reported suppression of neovascularization in both in vitro and in vivo models [23, 28]. This mechanism also supported by three studies which demonstrated that RSV intervention improved the retinal function in ERG [15, 19, 20].

### Drug administration and bioavailability

Resveratrol efficacy in preclinical diabetic retinopathy models is influenced by species and treatment duration, with prolonged systemic administration showing greater benefit in suppressing chronic neuroinflammation and mitochondrial dysfunction. In rodent models, daily doses of 10–50 mg/kg were most consistently associated with retinal protection, including reduced inflammatory cytokines, preserved blood–retinal barrier integrity, improved mitochondrial morphology, and functional recovery.

Treatment duration was a key determinant of outcome. Administration for ≥ 8 weeks, particularly at doses ≥ 10 mg/kg/day, produced more consistent neuroprotective and anti-inflammatory effects, whereas short-term treatment (< 4 weeks) resulted mainly in partial molecular changes with limited structural or functional improvement.

## Discussion

This systematic review synthesizes evidence from 20 preclinical studies and demonstrates that RSV exerts consistent antioxidant, anti-inflammatory, anti-angiogenic, and neuroprotective effects in DR models. Across in vitro and in vivo settings, RSV activated SIRT1, enhanced mitochondrial quality control, reduced pro-inflammatory cytokines (TNF-α, IL-6, IL-1β), and suppressed angiogenic mediators such as VEGF and TGF-β2.

Compared with earlier reviews that primarily summarized resveratrol’s general antioxidant and anti-angiogenic actions, this review provides an updated and integrative mechanistic synthesis emphasizing upstream regulatory pathways relevant to early diabetic retinopathy. A key novelty of this review is the integrated analysis of RSV’s effects on epigenetic regulators—including SIRT1, histone acetylation, and HMGB1—that drive microglial pro-inflammatory phenotypes. These findings support RSV as a multitarget compound capable of modulating upstream inflammatory and epigenetic pathways relevant to DR. Although effect sizes were not uniformly reported, the direction of changes was remarkably consistent across studies. This epigenetic modulation not only suppresses cytokine production but also promotes a shift from the M1 (pro-inflammatory) to the M2 (anti-inflammatory or reparative) phenotype—a finding primarily inferred from transcriptional patterns but supportive of RSV-induced microglial reprogramming as a potential early DR intervention.

**Fig. 4:**
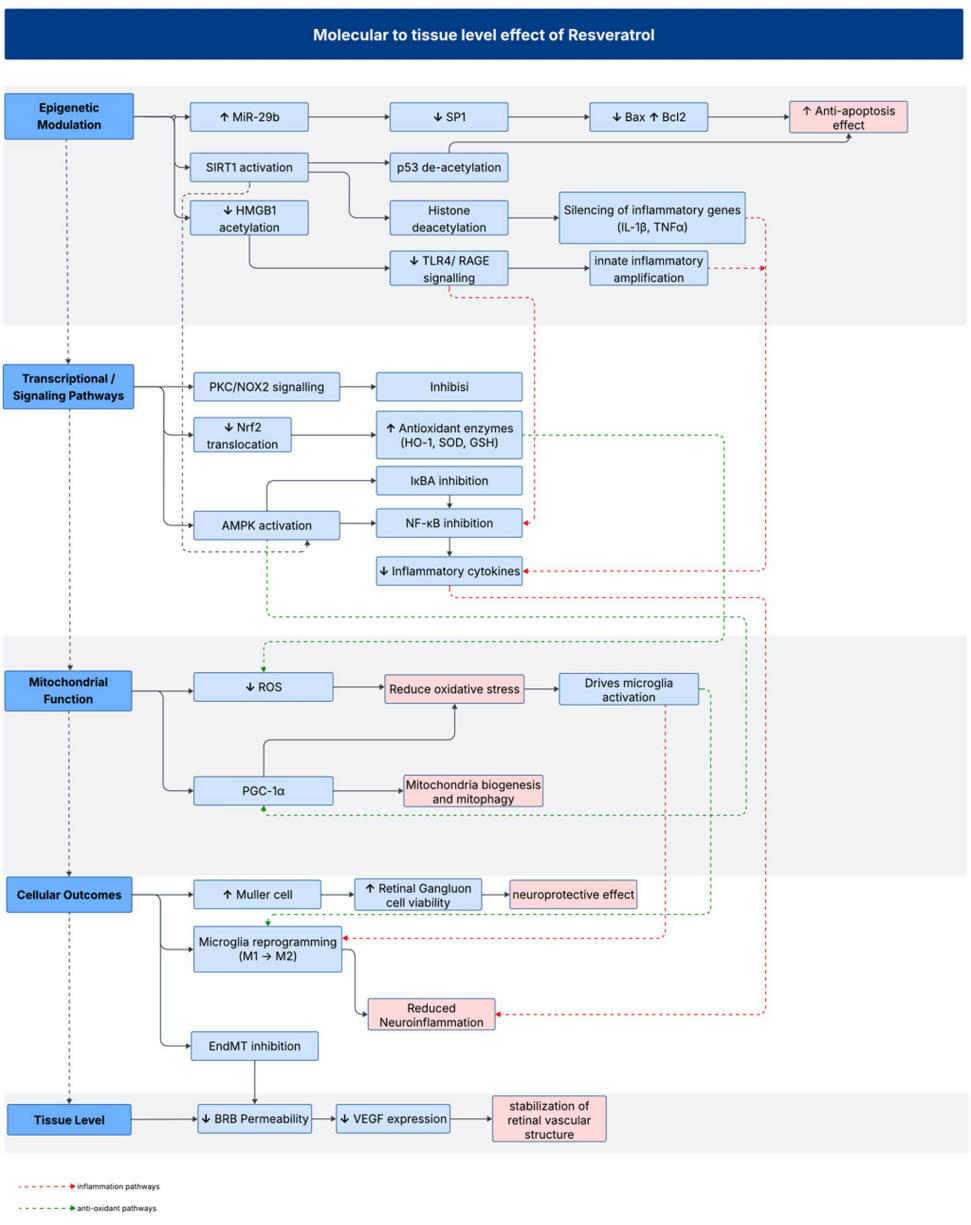
Molecular to tissue level effect of Resveratrol in Diabetics Retinopathy. Schematic overview of resveratrol’s multilevel effects in diabetic retinopathy, illustrating its roles in epigenetic regulation, anti-inflammatory signaling, mitochondrial protection, cellular reprogramming, and vascular stabilization from molecular to tissue level. RSV, resveratrol; DR, diabetic retinopathy; miR-29b, microRNA-29b; SP1, specificity protein 1; SIRT1, sirtuin 1; HMGB1, high mobility group box 1; TLR4, toll-like receptor 4; RAGE, receptor for advanced glycation end-products; IL-1β, interleukin-1 beta; TNF-α, tumor necrosis factor-alpha; PKC, protein kinase C; NOX2, NADPH oxidase 2; Nrf2, nuclear factor erythroid 2-related factor 2; HO-1, heme oxygenase-1; SOD, superoxide dismutase; GSH, glutathione; IκBα, inhibitor of NF-κB alpha; NF-κB, nuclear factor kappa B; AMPK, AMP-activated protein kinase; ROS, reactive oxygen species; PGC-1α, peroxisome proliferator-activated receptor gamma coactivator 1-alpha; BRB, blood-retinal barrier; VEGF, vascular endothelial growth factor; RGC, retinal ganglion cell; EndMT, endothelial-to-mesenchymal transition

This mechanistic framework is summarized in Fig. 4, which illustrates the molecular-to-tissue level effects of RSV in DR. RSV initiates SIRT1-mediated deacetylation-linked regulation, leading to histone deacetylation and suppression of inflammatory gene expression. This attenuates TLR4/RAGE signalling and limits innate immune amplification. Concurrently, RSV inhibits NF-κB nuclear translocation by stabilizing IκBα and activates downstream AMPK and Nrf2 pathways, thereby reducing oxidative stress and cytokine release across retinal cell types. At the cellular level, these upstream actions contribute to microglial reprogramming, suppression of HMGB1-driven DAMP signalling, and reduction of chronic neuroinflammation. RSV also supports mitochondrial integrity through the SIRT1/PGC-1α axis, limiting ROS-induced activation. Collectively, these mechanisms preserve blood-retinal barrier integrity, downregulate VEGF, and may prevent vascular leakage and neovascularization, particularly in early stages. Further translational studies are required to validate these mechanisms in human contexts.

Given its multitarget properties, RSV may have several clinical applications: (1) as an adjunct for incomplete anti-VEGF responders; (2) as preventive therapy for early DR by targeting oxidative and inflammatory dysregulation before microvascular damage occurs; and (3) as intravitreal or nanoparticle-enhanced formulations to improve retinal penetration. Importantly, RSV’s mechanisms differ from anti-VEGF therapy—which targets a single pathway and does not address early neuroinflammatory changes—positioning RSV as a complementary upstream-acting agent.

Furthermore, this review incorporates recent preclinical evidence over the last ten years. By synthesizing these emerging data, our review extends beyond descriptive summaries and proposes a conceptual model in which resveratrol acts as a multitarget, upstream modulator capable of reprogramming the inflammatory and metabolic retinal microenvironment during early disease stages.

Resveratrol is generally regarded as a safe compound and has been widely consumed as a dietary supplement. Evidence from clinical studies in non-ocular settings indicates that low oral doses ranging from 25 to 150 mg are generally well tolerated, with only mild adverse effects such as headache or dizziness reported in some individuals. At higher doses, particularly in the gram range (approximately 2.5–5 g/day), gastrointestinal symptoms including nausea, diarrhea, and abdominal discomfort have been observed. Importantly, severe systemic toxicity has not been consistently reported, supporting resveratrol’s overall favorable safety profile [12]. 

From a translational perspective, pharmaceutical development remains a critical challenge. Native resveratrol exhibits low aqueous solubility, rapid metabolism, and limited oral bioavailability, which restrict systemic and retinal exposure. To overcome these limitations, various formulation strategies—such as nanoparticle-based delivery systems, sustained-release platforms, and intravitreal formulations—have been explored in preclinical studies. These approaches have demonstrated improved tissue bioavailability and enhanced pharmacodynamic efficacy of polyphenolic compounds, suggesting that similar strategies may be required to optimize resveratrol-based ophthalmic therapeutics. However, standardized pharmaceutical-grade formulations, dose optimization, and long-term safety data specific to diabetic retinopathy remain to be established.

Despite promising findings, several gaps persist. Most available evidence derives from preclinical models, and direct human data remain limited, which constrains translational certainty. Although SIRT1 consistently emerges as a central regulatory node, its interactions with other transcriptional and metabolic regulators are not yet fully elucidated. Moreover, microglia-associated inflammatory modulation is frequently inferred from transcriptional or signaling changes rather than confirmed by direct phenotypic validation. Future studies incorporating immunohistochemistry or flow cytometry may provide more definitive evidence of resveratrol’s immunomodulatory effects within retinal tissue.

Methodological limitations should also be acknowledged. Many included studies demonstrated moderate risk of bias due to insufficient reporting of randomization procedures, blinding, and sample size calculations, potentially affecting internal validity and reproducibility. In addition, although reductions in inflammatory mediators and preservation of retinal structure were consistently reported, relatively few studies assessed functional visual outcomes such as electroretinography or behavioural measures. Long-term efficacy, durability of microglia-associated inflammatory modulation, and optimal dosing strategies therefore remain important areas for future investigation.

In conclusion, this systematic review consolidates growing evidence supporting resveratrol as a multi-targeted agent for DR management. Through SIRT1-mediated pathways, RSV modulates inflammation, prevents apoptosis, preserves mitochondrial and endothelial function, and protects retinal neurons, making it a compelling candidate for early-stage DR therapy. However, successful translation to clinical practice requires clarification of dosing strategies, long-term safety, and human efficacy. Integrating RSV into current treatment paradigms may shift DR management from reactive approaches to proactive neurovascular protection.

## Supplementary Information

Below is the link to the electronic supplementary material.

Supplementary Material 1

## Data Availability

The datasets used and/or analyzed during the current study are available from the corresponding author upon reasonable request.

## Abbreviations

AGE: Advanced Glycosylated End Product
AMPK: AMP-Activated Protein Kinase
ARPE-19: Adult Retinal Pigment Epithelial cell line-19
bFGF: Basic Fibroblast Growth Factor
BREC: Bovine Retinal Capillary Endothelial Cells
BRB: Blood-Retinal Barrier
CD31: Cluster of Differentiation 31
CDH5: Cadherin-5 (VE-cadherin)
CoCl₂: Cobalt(II) Chloride
COX-2: Cyclooxygenase-2
CTGF: Connective Tissue Growth Factor
DAMP: Damage-Associated Molecular Pattern
DM: Diabetes Mellitus
DR: Diabetic Retinopathy
EndMT: Endothelial-to-Mesenchymal Transition
ELISA: Enzyme-Linked Immunosorbent Assay
ERG: Electroretinography
ERK: Extracellular Signal-Regulated Kinase
GCL: Ganglion Cell Layer
GLAST: Glutamate Aspartate Transporter
GS: Glutamine Synthetase
GSH: Glutathione
GTPase: Guanosine Triphosphatase
HMGB1: High-Mobility Group Box-1
HO-1: Heme Oxygenase-1
HRCECs: Human Retinal Capillary Endothelial Cells
HRECs: Human Retinal Endothelial Cells
IFN-γ: Interferon Gamma
IL-1β: Interleukin-1 Beta
IL-6: Interleukin-6
INL: Inner Nuclear Layer
IκBα: Inhibitor of NF-κB Alpha
JNK: c-Jun N-terminal kinase
LPS: Lipopolysaccharide
MAPK: Mitogen-Activated Protein Kinase
MDA: Malondialdehyde
MCP-1: Monocyte Chemoattractant Protein-1
Mfn2: Mitofusin 2
miR: microRNA
NF-κB: Nuclear Factor Kappa B
NO: Nitric Oxide
NOX2: NADPH Oxidase 2
NQO1: NAD(P)H Quinone Dehydrogenase 1
Nrf2: Nuclear Factor Erythroid 2-Related Factor 2
ONL: Outer Nuclear Layer
OP: Oscillatory Potentials
PBMCs: Peripheral Blood Mononuclear Cells
PDR: Proliferative Diabetic Retinopathy
PGC-1α: Peroxisome Proliferator-Activated Receptor Gamma Coactivator 1-Alpha
PKC: Protein Kinase C
PON1: Paraoxonase 1
PTGS2: Prostaglandin-Endoperoxide Synthase 2 (COX-2)
qRT-PCR: Quantitative Real-Time Polymerase Chain Reaction
RAGE: Receptor for Advanced Glycation End-products
RAX: Retina and Anterior Neural Fold Homeobox
RGC: Retinal Ganglion Cell
RPE: Retinal Pigment Epithelium
RREC: Rat Retinal Endothelial Cell
ROS: Reactive Oxygen Species
RSV: Resveratrol
SIRT: Sirtuin
SM22: Smooth Muscle Protein 22-alpha
SOD: Superoxide Dismutase
SP1: Specificity Protein 1
STZ: Streptozotocin
TEM: Transmission Electron Microscopy
TGF-β2: Transforming Growth Factor-Beta 2
TLR4: Toll-Like Receptor 4
TNF-α: Tumor Necrosis Factor-Alpha
TUNEL: TdT-mediated dUTP Nick End Labelling
VCAM-1: Vascular Cell Adhesion Molecule-1
VEGF: Vascular Endothelial Growth Factor
vWF: von Willebrand Factor
