# Supplementary material for: Resveratrol as a multitarget modulator in diabetic retinopathy: a systematic review of in vitro and in vivo studies
Source: BMC Ophthalmol. 2026 Jan 29;26:55. doi: 10.1186/s12886-026-04623-0 (PMC12853864; doi:10.1186/s12886-026-04623-0)
Supplement: Supplementary file 1 — Supplementary Material 1 [file 12886_2026_4623_MOESM1_ESM.docx]

Supplement 1. Draft entry PubMED Search

| No | Entry | Filter | Total Findings |
| --- | --- | --- | --- |
| 1. | ("resveratrol"[MeSH Terms]) OR ("resveratrol"[Text Word]) | None | 19,447 |
| 2. | ("diabetic retinopathy"[MeSH Terms]) OR ("diabetic retinopathy"[Text Word]) | None | 44,898 |
| 3. | S1 AND S2 | None | 68 |
| 4. | S1 AND S2 | In the last 10 years | 53 |

| No | Entry | Filter | Total Findings |
| --- | --- | --- | --- |
| 1. | ("resveratrol"[MeSH Terms]) OR ("resveratrol"[Text Word]) | None | 19,888 |
| 2. | ("in vivo"[Text Word]) OR (in vivo study[Text Word]) | None | 1,171,543 |
| 3. | (("diabetes"[Text Word]) OR ("diabetic"[Text Word])) OR ("high glucose"[Text Word])) OR ("diabetic retinopathy"[Text Word]) | None | 948,261 |
| 3. | S1 AND S2 AND S3 | None | 209 |
| 4. | S1 AND S2 | In the last 10 years | 146 |

| No | Entry | Filter | Total Findings |
| --- | --- | --- | --- |
| 1. | ("resveratrol"[MeSH Terms]) OR ("resveratrol"[Text Word]) | None | 19,888 |
| 2. | ("in vitro"[Text Word]) OR (in vitro study[Text Word]) | None | 631,538 |
| 3. | (("diabetes"[Text Word]) OR ("diabetic"[Text Word])) OR ("high glucose"[Text Word])) OR ("diabetic retinopathy"[Text Word]) | None | 948,261 |
| 3. | S1 AND S2 AND S3 | None | 207 |
| 4. | S1 AND S2 | In the last 10 years | 207 |

Draft entry EBSCOhost Search

| No | Entry | Filter | Total Findings |
| --- | --- | --- | --- |
| 1. | SU "resveratrol" OR TX "resveratrol" | None | 73,086 |
| 2. | SU "diabetic retinopathy" AND TX "diabetic retinopathy" | None | 35,932 |
| 3. | S1 AND S2 | None | 212 |
| 4. | S1 AND S2 | In the last 10 years | 193 |

Draft entry ProQuest Search

| No | Entry | Filter | Total Findings |
| --- | --- | --- | --- |
| 1. | SU "resveratrol" OR TX "resveratrol" | None | 67,277 |
| 2. | SU "diabetic retinopathy" AND TX "diabetic retinopathy" | None | 112,728 |
| 3. | S1 AND S2 | None | 1.995 |
| 4. | S1 AND S2 | In the last 10 years | 1.731 |
